# *PIK3CA* mutation detection by circulating tumor cell sequencing guides the effective PI3K inhibitor treatment of a patient with hormone receptor-positive breast cancer transformed into triple-negative breast cancer: A case report

**DOI:** 10.1097/MD.0000000000048622

**Published:** 2026-05-15

**Authors:** Jing Zhang, Jing Pei, Guangxu Hao, Yao Meng, Fei Li, Heng Xu, Hong Liu

**Affiliations:** a Department of Breast Surgery, Xuzhou Hospital Affiliated to Jiangsu University (Xuzhou Cancer Hospital), Xuzhou, Jiangsu, People’s Republic of China; b Department of Breast Surgery, First Affiliated Hospital of Anhui Medical University, Hefei, Anhui, People’s Republic of China; c Nanjing RontiMed Laboratory Co., Ltd, Nanjing, Jiangsu, People’s Republic of China; d The Second Department of Breast Cancer, Tianjin Medical University Cancer Institute and Hospital, National Clinical Research Center for Cancer, Tianjin, China.

**Keywords:** breast cancer, case report, CDK4/6 inhibitor resistance, circulating tumor cells, phenotypic transformation, *PIK3CA* mutation, sequencing

## Abstract

**Rationale:**

Most patients with hormone receptor-positive breast cancer (BC) prefer endocrine therapy and chemotherapy. However, some HR+ BC patients often exhibit resistance to CDK4/6 inhibitors and even undergo molecular subtyping changes during disease progression. Therefore, precise detection and treatment of these patients after disease progression are crucial.

**Patient concerns:**

In October 2021, a 51-year-old Chinese female (Han ethnicity) with a palpable mass in her left breast was diagnosed with HR+ BC. After endocrine therapy and CDK4/6 inhibitor treatment, the patient’s disease still progressed and transformed into triple-negative BC.

**Diagnoses:**

Peripheral blood circulating tumor cells were isolated from the patient and performed targeted sequencing by using next-generation sequencing, revealing the presence of a *PIK3CA* mutation in the patient.

**Interventions:**

The patient’s condition was effectively controlled after treatment with PI3K inhibitors.

**Outcomes:**

The patient’s progression free survival was prolonged to 7 months after treatment with the PI3K inhibitor. All adverse reactions were tolerated after symptomatic treatment. Her adverse reactions remained tolerable until further progression.

**Lessons:**

Our case fully demonstrates the importance of the early detection of *PIK3CA* mutations in treatment strategies. In addition, circulating tumor cells-targeted sequencing technology could be used for the concomitant diagnosis of advanced BC and evaluate gene mutations under treatment pressure.

## 1. Introduction

Breast cancer (BC) has emerged as one of the most prevalent malignant tumors in women worldwide. The global cancer statistics for 2022 released by the International Agency for Research on Cancer revealed that BC is the second most prevalent cancer worldwide after lung cancer, ranking 4th in mortality rates and accounting for 2.3 million new cases.^[1]^ BC is a heterogeneous malignancy characterized by diverse molecular subtypes exhibiting distinct clinical characteristics and treatment responses. Hormone receptors (HRs) play an important role in BC development and progression.^[2]^ Approximately 70% of BC cases are HR-positive (HR+) BC.^[3–5]^ Their cells exhibit the positive expression of estrogen receptors (ERs), progesterone receptors (PRs), or both; these receptors are associated with the proliferation and spread of cancer cells. Estrogen and ERs are pivotal in the development and advancement of BC. PR is an upregulated target gene of ER, and its expression depends on estrogen. Notably, PR can modulate ER activity, serving as a valuable prognostic biomarker for overall survival or disease-free survival in BC.

The CDK4/6 inhibitors palbociclib, ribociclib, and abemaciclib are first-line treatments for recurrent unresectable BC, metastatic ER-positive (ER+) BC, or metastatic BC negative for human epidermal growth factor receptor 2 (HER2); in premenopausal women, they are used in combination with an aromatase inhibitor for ovarian function suppression.^[6]^ Data support the combination of a CDK4/6 inhibitor with the selective ER degrader fulvestrant, especially in patients experiencing progression or early relapse following adjuvant aromatase inhibitor therapy.^[7]^ Although CDK4/6 inhibitors enhance disease management in patients with HR+ BC, some patients do not exhibit a favorable response to these agents, and most patients whose tumors initially respond to CDK4/6 inhibitors eventually develop acquired resistance. Early and late adaptation facilitated by persistent G1–S-phase cyclin expression and alternative signaling pathways diminish the efficacy of CDK4/6 inhibitors.^[8,9]^

In this study, we examined a unique case of HR+ BC that progressed to triple-negative BC (TNBC) after resistance to a CDK4/6 inhibitor. The Chinese expert consensus on *PIK3CA* mutation testing in BC (2025 edition) recommends routine *PIK3CA* mutation testing prior to first-line therapy in patients with HR+/HER2-negative (HER2−) locally advanced or metastatic BC to guide the use of PI3K inhibitors.^[10]^ However, the consensus does not involve TNBC. Therefore, *PIK3CA* mutations in patients with TNBC have received little attention. Peripheral blood circulating tumor cell (CTC) sequencing revealed that our patient had a *PIK3CA* mutation. She then participated in a clinical trial on P13K inhibitors and experienced effective therapeutic effects.

## 2. Materials and methods

### 2.1. Patients

This study was performed in line with the principles of the Declaration of Helsinki. It was approved by the Ethics Committee of Xuzhou Cancer Hospital (approval date: October 21, 2024, approval code: 2024-CS-024). Informed consent forms were obtained from all patients and caregivers. Patients with advanced HR+ BC who were treated with CDK4/6 inhibitors between November 2024 to June 2025 were selected. Inclusion criteria included an initial diagnosis of HR+ BC and the emergence of CDK4/6 inhibitor resistance while the disease progressed to the advanced stage. Exclusion criteria included an initial diagnosis of TNBC or HER2-positive (HER2+) BC and the inability to tolerate endocrine therapy.

### 2.2. CTC separation and typing

The whole blood of patients with advanced HR+ BC who were treated with CDK4/6 inhibitors was used to isolate CTCs in accordance with the reagent instructions (HealthRun, Nanjing, China). In brief, 5 mL of peripheral blood was mixed with 15 mL of hemolytic agent, incubated at room temperature for 15 minutes on a rotary mixer at 10 rpm, and centrifuged at 700 × *g* for 15 minutes. The cell sediment was collected and resuspended. The sample was transferred to a filter column and sorted on a cell sorter (Weizhen, Suzhou, China). The remaining cells in the filter column were added with 12 μL of CD45 antibody-conjugated immunomagnetic beads, rotated, and reacted for 1 hour. The immunomagnetic beads were then separated by using a magnetic rack. The remaining liquid was transferred into a centrifuge tube and centrifuged at 300 × *g* at 25 °C for 10 minutes. The supernatant was removed, and approximately 100 μL of the sediment was retained at the bottom of the tube. After resuspension, the sample was dropped onto a glass slide and dried at 42 °C for 40 minutes. It was fixed for 5 minutes with 100 μL of fixative then rinsed. The sample was incubated with 100 μL of antibody dilution solution added with 1 μL each of panCK-AF488 fluorescent antibody, vimentin-AF568 fluorescent antibody, and CD45-AF647 fluorescent antibody at 37 °C in the dark for 1 hour. The sample was soaked and washed 3 times, dried at 37 °C in the dark for 5 minutes, then added with 10 μL of 4′,6-diamidino-2-phenylindole and gently covered with a cover glass. It was subsequently observed under a fluorescence microscope (Nikon, Japan).

### 2.3. DNA amplification

Multiple displacement amplification (MDA) is an amplification technology based on isothermal reactions. It utilizes φ29 DNA polymerase to amplify genomic DNA. By using Discover-sc Single Cell WGA Kit V2 (Catalog No. SC101, Vazyme, Nanjing, China), DNA was lysed and released from CTCs enriched from patient blood samples, then amplified on a PCR instrument (Dongshenglong, ETC821M) through MDA.

### 2.4. DNA whole exome library construction and sequencing

An enzyme digestion DNA library preparation kit (VAHTS Universal Plus DNA Library Prep Kit for Illumina V2,Vazyme, China) was used to fragment the DNA sample mentioned above and perform end repair, A addition to the 3′ end, linking, fragment sorting, and library amplification. The amplified library was purified by using magnetic beads, then its concentration was determined with Equalbit 1 × dsDNA HS Assay Kit (QE121, Vazyme, China) and a Qubit4 fluorescence quantitative analyzer (Thermo Fisher, Qubit 4.0).

One or several libraries were mixed in a certain proportion (calculated on the basis of the expected sequencing data volume, usually equal) in accordance with library concentration for pooling. A total of 500 ng of each library was collected for pooling (750 ng for a single library) and added with probes, buffers, blockers, and other components in accordance with instructions. The sample was reacted at 80 °C for 5 minutes in a PCR instrument and incubated at 50 °C for 12 to 16 hours to complete hybridization. After hybridization, the reaction products were washed and PCR amplified in accordance with instructions to complete WES hybridization and capture. After hybridization and capture, PE 150 sequencing was performed on the T7 sequencing platform (MGI, Huada, Shenzhen, China). The sequencing data volume of each sample was 12G, which is equivalent to a sequencing depth of 100×.

### 2.5. Sequencing data analysis

Quality assessment was conducted on the raw FASTQ files generated by sequencing. FastQC was used to detect sequence quality score distribution, GC content, joint contamination, and repeat sequence ratio. Multiple-sample QC results were integrated by using MultiQC. Cutadapt or Trimmomatic was employed for joint resection and low-quality sequence trimming. Common QC indicators included total reads, Q30 base ratio (usually > 80%), whether the GC content meets expectations, and sequence duplication ratio. High-quality clean reads were aligned to the human reference genome hg38 by using BWA. The average sequencing depth was maintained at >500× for targeted regions to ensure the high sensitivity of variant detection. Variant calling was performed with GATK, and variants were annotated by employing ANNOVAR. Variants were filtered on the basis of the following criteria to minimize false positives: sequencing depth ≥100× at the variant site and variant allele frequency ≥1% for somatic mutation analysis.

High-quality reads were aligned to the main reference genome GRCh37 or GRCh38, without or only containing the main assembly. BWA-MEM was utilized as the comparison tool. SAM/BAM format files were generated after the comparison was completed. Samtools was employed to sort and index BAM files. Picard MarkDuplicates were applied to label PCR repeat reads. After duplicates were labeled, sequencing coverage and coverage uniformity were counted again by using Qualimap and Picard CollectHsMetrics.

Subsequent data cleaning was performed with reference to GATK best practices. The corrected BAM call mutation was used. For candidate mutations, a priority list was established and pathogenicity was classified in accordance with ACMG/AMP guidelines. A subset of clinically relevant mutations was further validated by using independent methods, such as Sanger sequencing or droplet digital PCR, to ensure the accuracy of detected variants. Only mutations consistently detected across platforms were considered as true positive events. In addition, common germline variants were excluded through comparison with public databases, including dbSNP, 1000 Genomes Project, and ExAC.

The final output format included annotated VCF files and summary tables (CSV/Excel/PDF). The VCF format is a standard variant data output format that includes information, such as genome location, gene/transcript variants, genotype quality, depth, and annotations, for each variant.

## 3. Results

A total of 27 CDK4/6 inhibitor–resistant patients with HR+ BC (age: 56.2 ± 13.5 years), including 8 grade III and 19 grade IV patients, were enrolled for CTC sequencing. Table 1 summarizes the frequency (>3 times) of major gene mutations detected by CTC sequencing in these patients. CTC sequencing revealed that *TP53*, *BRCA1/2*, *PTEN*, and *PIK3CA* mutations occurred frequently in patients with advanced HR+ BC and CDK4/6 inhibitor resistance. Adjustments to the therapeutic schedule for specific patients could be made on the basis of these gene mutations. CTC sequencing showed that all patients had >1 gene mutation. Of the 8 patients with the *PIK3CA* mutation, 1 voluntarily participated in a clinical trial of P13K inhibitors. We review the disease development and therapy of this patient (Table 2).

**Table 1:** Summary of the frequency (>3 times) of major gene mutations detected by CTC sequencing in 27 patients with HR+ BC.

Mutated genes	Frequency	Potential clinical significance
EGFR	7	Related to targeted therapy
ERBB4	4	Related to tumor occurrence and development
TP53	16	Related to tumor occurrence and development
KMT2C	7	Related to tumor occurrence and development
BRCA1/2	18	Related to targeted therapy
PTEN	12	Related to targeted therapy
MET	4	Related to tumor occurrence and development
PIK3CA	8	Related to targeted therapy
ERBB2	6	Related to tumor occurrence and development
NRAS	5	Related to targeted therapy

CTC sequencing showed that all patients had more than one gene mutation.

CTC = circulating tumor cell.

**Table 2:** Disease treatment of the patient with HR+ BC and the PIK3CA mutation.

Time	Age (years old)	Progression	Therapeutic schedule	PFS (Months)	*PIK3CA* mutation
October 2021	51	First visit; PR	Operation; endocrine therapy		NA
November 2024	54	The first PD; pulmonary metastasis	RuiboXili combinated with fulvestrant	36	NA
March 2025	55	The second PD; multiple metastases	RuiboXili combinated with fulvestrant	3	CFM
November 2025	55	PD again	PI3K inhibitor	7	CFM

CFM = PIK3CA mutation confirmed but CTC sequencing, CTC = circulating tumor cell, NA = not tested, PD = progressive disease, PFS = the patient begins treatment and disease progression is observed, PR = partial response.

**Figure 1.**
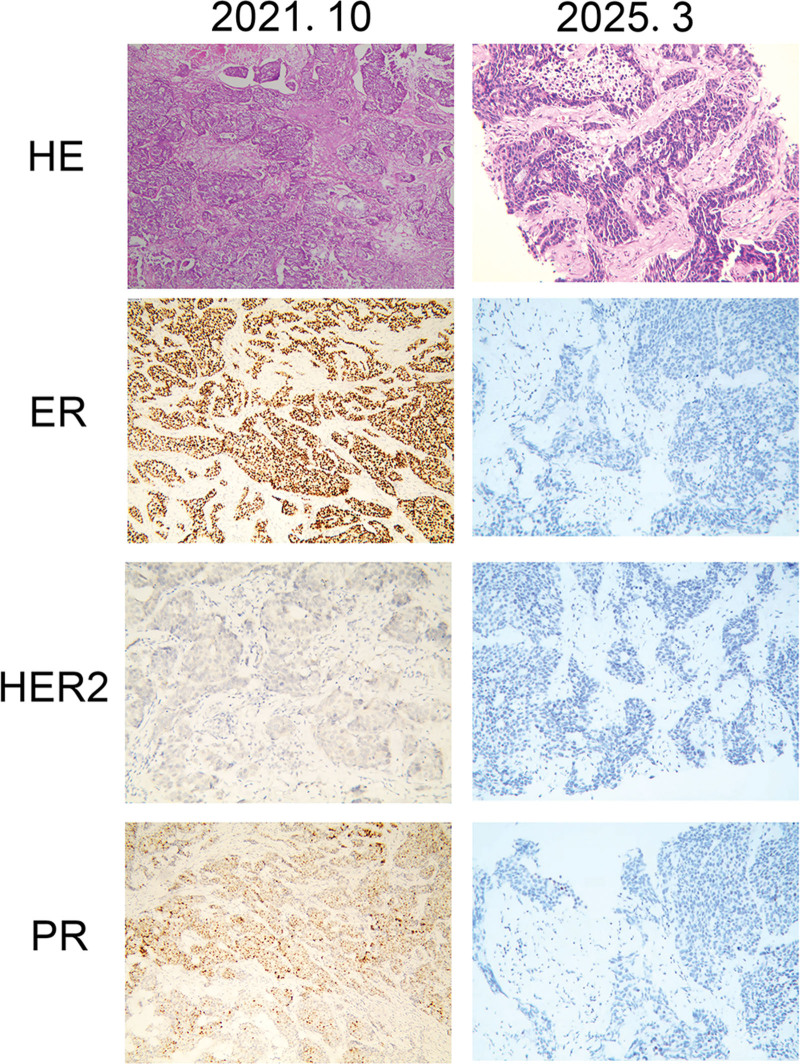
Pathological and immunohistochemical results of tissues from the patient in October 2021 and March 2025.

A 51-year-old female presented in October 2021 with a palpable mass in her left breast. Pathological examination indicated invasive ductal carcinoma (grades II–III) with axillary lymph node metastasis (3/15). Immunohistochemistry revealed ER+++ (40%), PR+++ (90%), HER2 (1+), and Ki67 (70%), indicative of HR+/HER2− BC (Fig. 1). The patient received surgical intervention, subsequently undergoing chemotherapy and radiotherapy.

**Figure 2.**
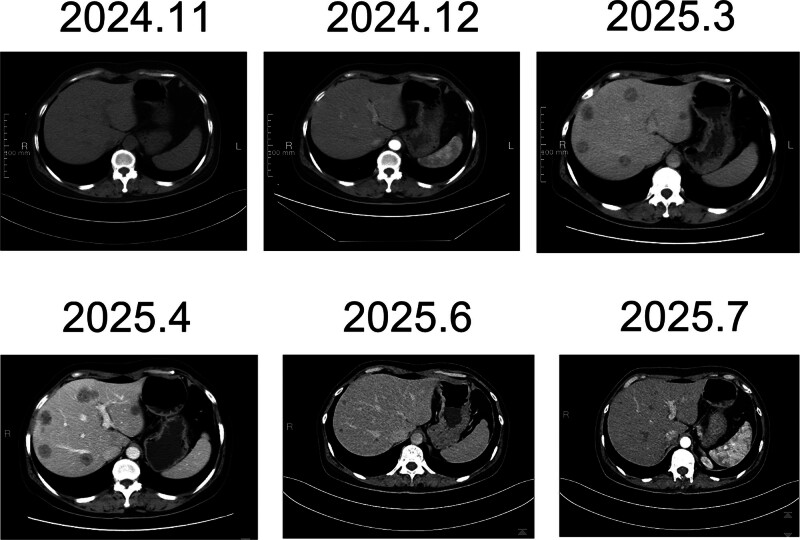
CT diagnoses of the patient during treatments. CT = computed tomography.

In August 2022, she participated in a clinical trial comparing treatment combining dalpiciclib and letrozole with letrozole monotherapy. On her 2-year follow-up in August 2024, PET–computed tomography (CT) revealed supraclavicular and mediastinal lymphadenopathy, along with a 6 mm × 7 mm pulmonary nodule in the right upper lobe exhibiting mildly increased uptake, indicative of metastasis (Fig. 2). She met the criteria for primary endocrine resistance.

She was transitioned to ribociclib in combination with fulvestrant. After 3 months (November 2024), a follow-up CT revealed disease progression, which was indicated by an 11 mm × 9 mm nodule in the right upper lung and >30% increase in size (Fig. 2). Additionally, the patient met the RECIST criteria for progressive disease, and primary resistance to CDK4/6 inhibition was confirmed.

**Figure 3.**
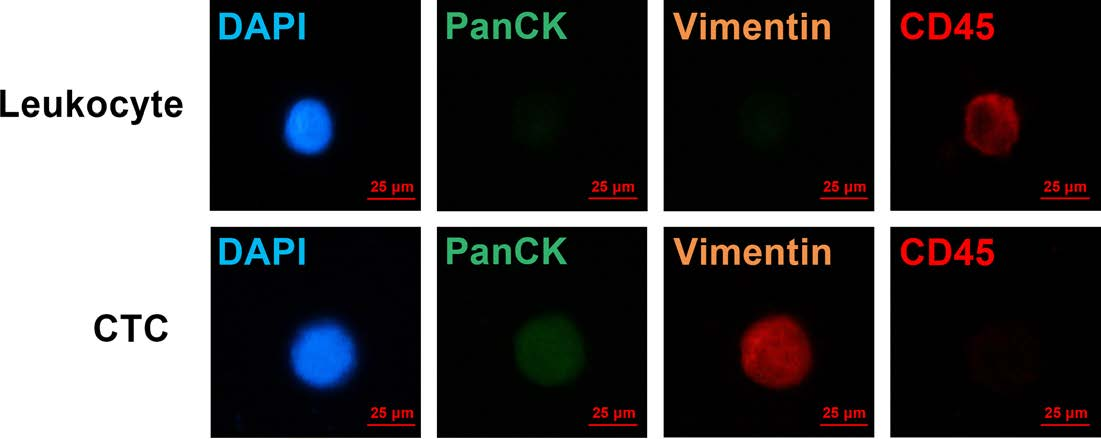
CTC microscopic examination by IFA. CD45 antibody labeled with AF647 (deep red). PanCK antibody labeled with AF488 (green). Vimentin antibody labeled with AF568 (orange-red). DAPI (blue). CTC = circulating tumor cell, DAPI = 4′,6-diamidino-2-phenylindole.

In December 2024, she participated in our study, which employed peripheral blood CTC sequencing for genomic variation analysis. A total of 12 CTCs were detected, including 0 epithelial CTCs, 0 interstitial CTCs, and 12 mixed epithelial–interstitial types (Fig. 3). The next-generation sequencing (NGS) of CTCs identified a missense mutation in *PIK3CA* (exon 21: c.G3129A:p.M1043I) and PTEN (exon 5: c.G395A:p.G132D) (Table 3).

**Table 3:** Summary of the CTC detection and sequencing results of the patient.

Detection	Results	
CTC counting and classification	A total of 12 CTCs were detected, including 0 epithelial CTC, 0 interstitial CTC, and 12 mixed epithelial-interstitial type	
CTC genomic variation	With clinical significance for targeted therapy	PTEN:exon5:c.G395A:p.G132D (missense mutation) PIK3CA:exon21:c.G3129A:p.M10431 (missense mutation)
	Related to tumor occurrence and development	KMT2C:exon16:c.G2722T:p.G908C (missense mutation) MYC:exon2:c.142 144del:p.Q52del (loss-of-function mutation) TP53:exon9:c.G957T:p.K319N (missense mutation)
	MSI detection	MSS
	PD-L1 efficacy positively correlated gene variation	TP53:exon9:c.G957T:p.K319N (missense mutation)
	PD-L1 efficacy negatively correlated gene variation	No detection

CTCs = circulating tumor cells.

Our patient persisted with the same treatment until March 2025. Subsequent chest and abdominal CT scans indicated multiple liver lesions suggestive of metastasis (Fig. 2). The biopsy of the right upper lobe nodule confirmed metastatic invasive ductal carcinoma originating from the breast. Immunohistochemistry revealed ER+ (2%), PR−, HER2−, and Ki67 (60%), indicating a TNBC phenotype (Fig. 1). Our patient then participated in a clinical trial for a PI3K inhibitor developed by Jiayue Biotech. After 2 months of treatment, a CT scan on June 4, 2025, revealed a reduction of >30% in the size of the target lesion, indicating a partial response. Our patient continues therapy with ongoing monitoring. In consideration of the results of CTC sequencing, we detected *PIK3CA* mutations through NGS with the patient’s postoperative tissue, and our results showed that a *PIK3CA* mutation was already present in postoperative tissue obtained in October 2021 (Table 4).

**Table 4:** *PIK3CA* mutation information of the patient with BC.

Detecting subject	VAF	Sequencing depth	Transcript and exon	Code DNA sequence	Variant site	Mutation type
CTC	2.3%	3200×	NM_006218:exon21	C.G3129A	p.M1043I	Missense mutation
Tissue	33.7%	850×	NM_006218:exon21	C.A3140G	p.H1047R	Missense mutation

CTCs: peripheral blood was collected in December 2024 for circulating tumor cell (CTC) isolation, followed by NGS to identify genomic alterations. Tumor tissue: genomic variation analysis was performed using NGS on tumor tissue collected in October 2021.

CTCs = circulating tumor cells; VAF = variant allele frequency.

We also followed up on our patient during treatment with the PI3K inhibitor. Her progression free survival was prolonged to 7 months after treatment with the PI3K inhibitor. By comparison, the median progression free survival for advanced TNBC multiline chemotherapy is 1 to 2 months. During the first 2 months of treatment, our patient experienced symptoms, such as fatigue, nausea, decreased appetite, bone marrow suppression, and stomatitis. These adverse reactions were tolerated after symptomatic treatment. Her adverse reactions remained tolerable until further progression.

## 4. Discussion

PR, ER, and HER2 are commonly employed for BC typing and therapeutic direction.^[11]^ The ineffectiveness of BC treatment is due to the variability in mutated genes and differences among patients. The pathogenesis of BC is not fully elucidated, and the risk factors affecting its incidence vary.^[12]^ A multicenter study of subtype switching in BC brain metastases showed that estrogen and progesterone determine hormonal status; 50 of 219 patients (22.8%) exhibited subtype transitions, of which 20 are based on HER2.^[13]^ In the present study, the molecular phenotype transformation of our patient with BC during treatment may be a complex phenomenon caused by tumor heterogeneity.^[14]^ BC tissue may exhibit spatial heterogeneity (varying molecular characteristics in different regions) or temporal heterogeneity (occurrence of gene mutations over time). Receptor-positive areas may be detected during the initial detection, whereas triple-negative clones dominate in recurrent or metastatic lesions, leading to a diagnosis of molecular subtype transition.^[15]^ The molecular characteristics of recurrent or metastatic lesions may differ from those of primary lesions. Under treatment pressure, the originally small-triple negative clone is selectively amplified, resulting in the recurrence of the focus showing TNBC.^[16]^ In clinical practice, transitions from ER+ to ER negative are not uncommon and may be associated with epigenetic regulation. In response to treatment, cancer cells turn off genes that express ERs. Moreover, the downregulation of ER expression under therapeutic pressure may contribute to the loss of HR expression.^[17]^ A study suggested that HER2-targeted therapy may induce luminal cells to acquire basal-like features,^[18]^ and phenotype transformation has been found to be correlated with the immune microenvironment. Classification conversion directly affects the selection of treatment plans.

*PIK3CA* mutations, a major genetic driver in BC, are commonly detected in this malignancy.^[19,20]^ The mutation rates of *PIK3CA* in luminal A BC, luminal B BC, HER2+ BC, and TNBC are 47%, 33%, 23% to 39% and 8% to 25%, respectively.^[21–23]^ Additionally, *PIK3CA* mutations are common in Chinese patients with BC.^[10]^ Some PI3K inhibitors have been approved by regulatory bodies for the treatment of specific patient populations with BC, and many new PI3K inhibitors have shown great promise for cancer treatment.^[24]^ Alpelisib, a selective p110α inhibitor, has been approved for the treatment of HR+/HER2− PIK3CA-mutant metastatic BC that has progressed after first-line endocrine therapy. *PIK3CA* mutations are present in TNBC and HER2+ BC. However, the effectiveness of PI3K inhibition in these subtypes is not yet clearly established.^[25]^ Combinations of PI3K and CDK4/6 inhibitors have demonstrated antitumor efficacy across multiple cancer types, with efficacy observed in preclinical models of *PIK3CA*-mutant TNBC.^[26]^ The activation of the PI3K/AKT/mTOR pathway is a known resistance mechanism in endocrine and CDK4/6 therapies; however, cases of HR+ BC evolving into TNBC with a *PIK3CA* mutation subsequent to resistance to CDK4/6 inhibitors are rare. Patients with HR+ BC that progressed to TNBC benefits from treatment with PI3K inhibitors. The relationship between *PIK3CA* mutations and phenotypic transformation may be demonstrated by the activation of the PI3K pathway by mutations that downregulate ER expression. Interestingly, in our study, the loss or silencing of the phenotypic inheritance of our patient with BC under treatment pressure led to transformation into HR− cancer. This phenomenon did not change the fact of *PIK3CA* mutation. In addition, the results of tissue and CTC sequencing showed that the mutation site of *PIK3CA* in our patient with BC changed after molecular subtyping transformation. The change in the *PIK3CA* mutation site is the result of the random accumulation of gene variations, which are mainly caused by DNA replication errors, environmental mutagenic factors, or endogenous damage, by somatic cells during tumor occurrence and development and does not directly lead to the transformation of BC molecular typing. It is commonly present as an intrinsic feature of specific molecular subtypes (especially HR+/HER2− types), reflecting the abnormal activation of signaling pathways within tumors.^[16,27]^

Recently, NGS using ctDNA and CTCs has been used to detect *PIK3CA* mutations in BC.^[28,29]^ Liquid biopsy can be performed through CTCs because they more precisely represent the genetics of metastatic tumors than that of primary tumors. Sanger sequencing using CTCs isolated from metastatic BC detected a pathogenic *PIK3CA* mutation in 2 out of 11 (18%) cases. Therefore, CTC analysis may be used to identify mutations in patients and tumors and cancer cells that are likely to metastasize.

An interesting finding regarding our case is that the *PIK3CA* mutation in our patient was confirmed through CTC sequencing. Her health had already considerably improved. However, her molecular subtype was initially HR+ BC. Additionally, no testing for *PIK3CA* mutations was performed after surgery. After finding the *PIK3CA* mutation through CTC sequencing, we conducted *PIK3CA* detection using postoperative tumor tissues. Our patient’s molecular subtyping was HR+ BC, which indicated a *PIK3CA* mutation. This result indicates that *PIK3CA* mutations may arise concurrently with disease progression and are unaffected by molecular subtyping changes. The early detection of *PIK3CA* mutations can contribute to the development of therapeutic schedules. In addition, CTC-targeted sequencing can serve as a complementary diagnostic method for detecting *PIK3CA* mutations, thereby enabling prompt adjustments to treatment.

We present a case of HR+ BC that initially showed resistance to CDK4/6 inhibitors and then progressed to TNBC. CTC sequencing discovered a *PIK3CA* mutation, demonstrating the potential effectiveness of using PI3K inhibitors. The discovery of *PIK3CA* mutations provides essential information for the future treatment of patients. The case report demonstrated the effectiveness of PI3K inhibitor therapy in a patient with a *PIK3CA* mutation and a molecular classification that changed from HR+ BC to TNBC after the development of resistance to CDK4/6 inhibitors. CTC sequencing played a pivotal role in identifying resistance mechanisms and guiding precision therapy. The case supports the incorporation of dynamic rebiopsy and molecular surveillance into conventional clinical practice for the management of resistance.

Our study has several limitations. First, it only described a single patient, and its small sample size cannot be statistically analyzed, making its results difficult to extrapolate. Second, *PIK3CA* genomic changes were not continuously tracked, and the lack of longitudinal genomic monitoring makes capturing dynamic molecular changes under disease progression, treatment resistance, or treatment pressure impossible. Third, our study lacked a control group. The absence of comparative molecular data (such as genomics data) can make distinguishing between therapeutic effects and individual baseline variations difficult, complicating establishing causal relationships.

## 5. Conclusion

This clinical trial of PI3K inhibitors mainly focuses on patients with HR+/HER− BC, and the clinical study on patients with TNBC and *PIK3CA* mutations is ongoing. The case reported herein provides evidence that PI3K inhibitors can also be used to treat patients with TNBC and *PIK3CA* mutations. Attention should be paid to *PIK3CA* mutations because patients with BC can benefit from PI3K inhibitor treatment regardless of their classification as HR+ BC, TNBC, or BC with molecular subtyping changes.

## Acknowledgments

The authors gratefully acknowledge MD Jing Zhuo, Chief Physician, Deputy Director of the Pathology Department of Xuzhou Cancer Hospital, for their valuable input.

## Author contributions

**Conceptualization:** Jing Zhang, Hong Liu.

**Data curation:** Yao Meng.

**Funding acquisition:** Jing Zhang.

**Investigation:** Jing Pei, Fei Li.

**Methodology:** Guangxu Hao, Heng Xu.

**Writing – original draft:** Heng Xu.

**Writing – review & editing:** Jing Zhang.
